# Overcoming Missing Data: Accurately Predicting Cardiovascular Risk in Type 2 Diabetes, A Systematic Review

**DOI:** 10.1111/1753-0407.70049

**Published:** 2025-01-22

**Authors:** Wenhui Ren, Keyu Fan, Zheng Liu, Yanqiu Wu, Haiyan An, Huixin Liu

**Affiliations:** ^1^ Department of Clinical Epidemiology and Biostatistics Peking University People's Hospital Beijing China; ^2^ Department of Anesthesiology Peking University People's Hospital Beijing China

**Keywords:** cardiovascular diseases, data handling, risk assessment, statistical data interpretation, statistical model, type 2 diabetes mellitus

## Abstract

Understanding is limited regarding strategies for addressing missing value when developing and validating models to predict cardiovascular disease (CVD) in type 2 diabetes mellitus (T2DM). This study aimed to investigate the presence of and approaches to missing data in these prediction models. The MEDLINE electronic database was systematically searched for English‐language studies from inception to June 30, 2024. The percentages of missing values, missingness mechanisms, and missing data handling strategies in the included studies were extracted and summarized. This study included 51 articles published between 2001 and 2024, involving 19 studies that focused solely on prediction model development, and 16 and 16 studies that incorporated internal and external validation, respectively. Most articles reported missing data in the development (*n* = 40/51) and external validation (*n* = 12/16) stages. Furthermore, the missing data were addressed in 74.5% of development studies and 68.8% of validation studies. Imputation emerged as the predominant method employed for both development (27/40) and validation (7/12) purposes, followed by deletion (17/40 and 4/12, respectively). During the model development phase, the number of studies reported missing data increased from 9 out of 15 before 2016 to 31 out of 36 in 2016 and subsequent years. Although missing values have received much attention in CVD risk prediction models in patients with T2DM, most studies lack adequate reporting on the methodologies used for addressing the missing data. Enhancing the quality assurance of prediction models necessitates heightened clarity and the utilization of suitable methodologies to handle missing data effectively.

## Graphical abstract

Missing data handling approach at development stage of prediction model.

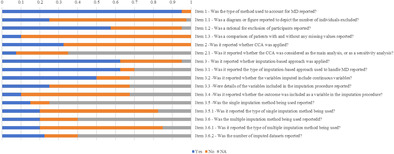

**Summary**

- In this systematic review of 51 studies, missing value were handled in 74.5% (38/51) of developmental studies and 68.8% (11/16) of validation studies.
- Imputation emerged as the predominant missing data handling method employed for both development and validation purposes, followed by deletion.
- While missing values in CVD prediction models for patients with T2DM have been studied, better clarity and appropriate methodologies for addressing missingness are still needed to improve prediction model quality.

## 1 Introduction

Type 2 diabetes mellitus (T2DM) ranks as the eighth most prevalent cause of mortality and morbidity, emerging as a significant global public health issue [1]. Cardiovascular disease (CVD) continues to be the leading cause of death and disability in individuals with T2DM [2]. Accumulating evidence shows that patients with T2DM exhibit a 2–4 times greater likelihood of developing CVD than those without T2DM, resulting in a higher disease burden, hospitalization, and treatment costs [3, 4]. Therefore, timely identification of individuals at high risk for CVD is essential for initiating early prevention strategies, providing prompt treatment, and ensuring effective disease management. Various risk prediction models have been developed and validated for CVD in patients with T2DM [5, 6, 7]. However, the productive performance and quality of such models are uneven and depend heavily on the quality and availability of datasets.

Missing data is a pervasive and significant challenge during the development of prediction models, especially when utilizing extensive datasets. This missingness can result in biased estimations, diminished statistical power, and inaccurate conclusions, thereby compromising the predictive performance of the model and diminishing its decision‐making efficacy in clinical practice. The Transparent Reporting of a multivariable prediction model for the Individual Prognosis or Diagnosis (TRIPOD) initiative has been proposed. It recommends that authors transparently report the occurrence and extent of missing values in both the development and validation sets, as well as detail the methods used to address any missing data during the analysis [8]. Thus, other researchers and policymakers must adequately assess the potential usefulness of prediction models. However, compliance with these reporting guidelines appears to be constrained among studies that apply prediction models, particularly when missing data are often inadequately addressed or disregarded.

Multiple approaches have been developed to address missingness, including complete case analysis (CCA) and imputation‐based methods. These strategies are always valid under specific circumstances [9, 10]. Moreover, many reviews of prediction models have found that the approaches for addressing missing values are inadequately explained or exhibit unclear applications [11]. A recent review examined the patterns of missing data and strategies for addressing them in predictive research for undiagnosed T2DM, while the handling of missing data in prediction models for CVD among individuals with T2DM remains unexplored [12].

Therefore, this study aimed to systematically review the prediction model of CVD for individuals with T2DM as the primary outcome and analyze the presence of missing data, missing scenarios, and the application of different strategies for processing missing data.

## 2 Methods

### 2.1 Literature Search

The search strategy was designed for the MEDLINE electronic database from its inception until June 30, 2024, using variations of search terms including “diabetes mellitus type 2,” “cardiovascular diseases,” and “prediction model” (Supporting Information: Appendix). The references cited in the articles were meticulously examined using the snowballing approach to identify additional relevant research.

### 2.2 Study Selection

**FIGURE 1:**
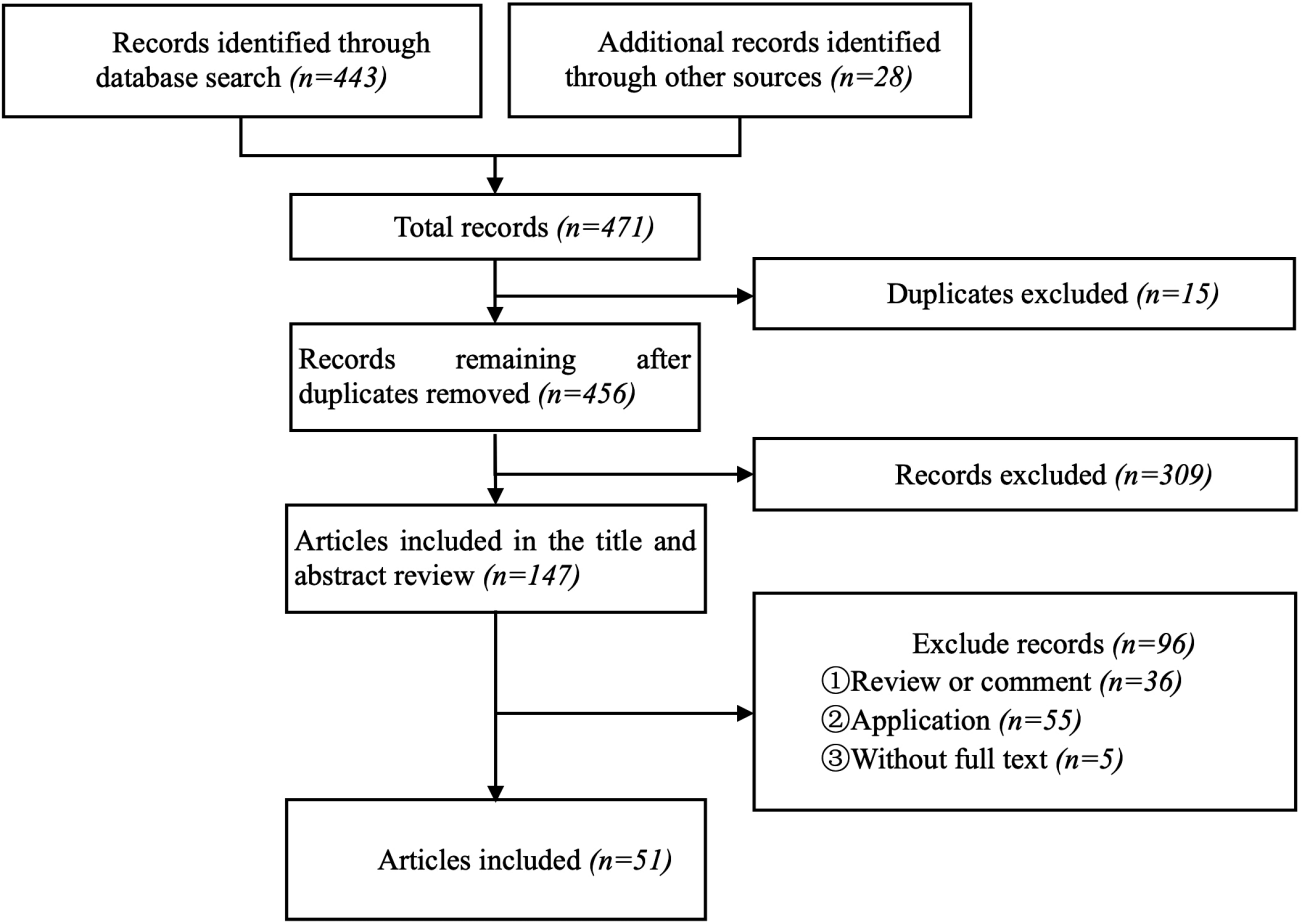
Flow diagram of Preferred Reporting Items for Systematic Reviews and Meta‐Analyses.

Figure 1 displays this study's Preferred Reporting Items for Systematic reviews and Meta‐Analyses flow diagram. The following eligibility criteria for studies were applied: (1) patients diagnosed with T2DM; (2) studies focused on developing prediction models; (3) outcome(s) of the predictive model was CVD or included CVD; and (4) English‐language. Reviews, studies that only reported the external validation stage, and papers for which full‐text versions were unavailable were excluded. Two reviewers (W.H.R. and K.Y.F.) independently reviewed the article titles and abstracts. Discrepancies were evaluated by another reviewer (H.X.L).

### 2.3 Data Extraction and Analysis

A standardized data extraction template was developed to catalogue basic publication information (i.e., publication year and study design) and the characteristics of the predictive models (i.e., data source, study population description, sample size, outcomes, statistical models, and predictive model stage). Whether and how well the included studies reported on and addressed the issue of missing data were also summarized based on previous studies [8], which included information missingness of the study variables (i.e., missingness mechanisms and percentage of missing values) and missing data handling strategies (i.e., deletion, imputation‐based methods, and non‐imputation‐based methods).

## 3 Results

The preliminary literature search identified 471 articles. After removing duplicate entries and applying the inclusion and exclusion criteria, 51 articles were included for further review (Figure 1).

### 3.1 Study Characteristics

Table S1 presents an overview of the characteristics of the 51 studies that were included in the analysis, which were published between 2001 and 2024 [5, 13, 14, 15, 16, 17, 18, 19, 20, 21, 22, 23, 24, 25, 26, 27, 28, 29, 30, 31, 32, 33, 34, 35, 36, 37, 38, 39, 40, 41, 42, 43, 44, 45, 46, 47, 48, 49, 50, 51, 52, 53, 54, 55, 56, 57, 58, 59, 60, 61, 62]. Many studies (19 of 51) focused only on the development process for the prediction models [15, 18, 19, 21, 22, 23, 24, 25, 31, 35, 46, 48, 49, 52, 55, 56, 57, 59, 61], 16 articles on model development also included the internal validation stage [13, 16, 20, 28, 32, 33, 34, 36, 37, 40, 43, 45, 51, 54, 60, 62], and 16 articles conducted additional external validation [5, 14, 17, 26, 27, 29, 30, 38, 39, 41, 42, 44, 47, 50, 53, 58]. The study sample sizes regarding the developmental stages of the prediction models varied from 151 to 1 297 131.

Cohort studies comprised 39 of the articles included in this review [5, 13, 14, 15, 18, 20, 21, 22, 24, 26, 27, 28, 30, 31, 32, 33, 34, 36, 37, 39, 42, 43, 44, 45, 46, 47, 48, 49, 51, 53, 54, 55, 56, 57, 58, 59, 61, 62], while six [16, 17, 23, 40, 59, 60] and eight studies [19, 25, 29, 35, 38, 41, 50, 52] developed risk prediction models based on cross‐sectional studies and randomized controlled trials, respectively. Accordingly, seven studies focused on developing diagnostic prediction models [25, 26, 35, 40, 44, 47, 59], while the remaining studies primarily centered on prognostic prediction models [13, 14, 15, 16, 17, 18, 19, 20, 21, 22, 23, 24, 27, 28, 29, 30, 31, 32, 33, 34, 36, 37, 38, 45, 46]. Regarding model types, most studies (35/51) applied the Cox proportional hazards regression model [13, 14, 15, 17, 18, 19, 20, 22, 24, 25, 27, 28, 29, 30, 31, 33, 34, 35, 36, 38, 41, 42, 45, 48, 49, 50, 51, 52, 53, 54, 55, 56, 57, 58, 61], followed by a logistic regression model (10/51) [16, 23, 26, 32, 37, 39, 40, 43, 44, 59]. The other models included decision tree [24, 39, 44], knowledge learning symbiosis [46], random survival forest methods [47], and deep neural network [60].

### 3.2 Presence and Mechanisms for Handling Missing Data

Almost 78.4% (40/51) of the included studies reported missing values at the prediction model development stage [5, 13, 14, 15, 16, 17, 18, 20, 21, 24, 26, 27, 29, 30, 32, 33, 35, 36, 37, 39, 40, 41, 42, 43, 44, 45, 46, 47, 48, 49, 50, 51, 52, 53, 54, 56, 57, 58, 61, 62] while 75% (12/16) of studies reported missingness in the external validation stage [5, 14, 26, 27, 30, 39, 41, 42, 44, 50, 53, 58]. Additionally, 22 studies reported the percentage of missing data [5, 14, 17, 20, 21, 26, 29, 32, 33, 39, 40, 42, 43, 44, 46, 47, 50, 51, 52, 53, 54, 58, 61] that most often included the overall number (*n* = 6) [29, 42, 43, 44, 51, 52] or frequency of missingness (*n* = 16) [5, 14, 17, 20, 21, 26, 32, 33, 39, 40, 46, 47, 53, 54, 58, 61]. The extent of missingness ranged from < 1% to 97.81%. Only four studies described the mechanisms of missing data, specifically addressing data missing at random (MAR) (*n* = 2) and missing not at random (MNAR) (*n* = 2) [15, 17, 40, 58] (Table S2).

### 3.3 Strategies for Handling Missing Data

A total of 38 out of 40 articles that reported missing values also included a description of the strategies applied to handle missing data [5, 13, 14, 15, 16, 17, 18, 20, 21, 26, 27, 29, 30, 32, 33, 35, 36, 37, 39, 40, 42, 43, 44, 45, 46, 47, 48, 49, 50, 51, 52, 53, 54, 56, 57, 58, 61, 62], and 11 studies consisting of an external validation stage [5, 14, 26, 27, 30, 39, 42, 44, 50, 53, 58] (Table S2).

#### 3.3.1 Development Stage

**FIGURE 2:**
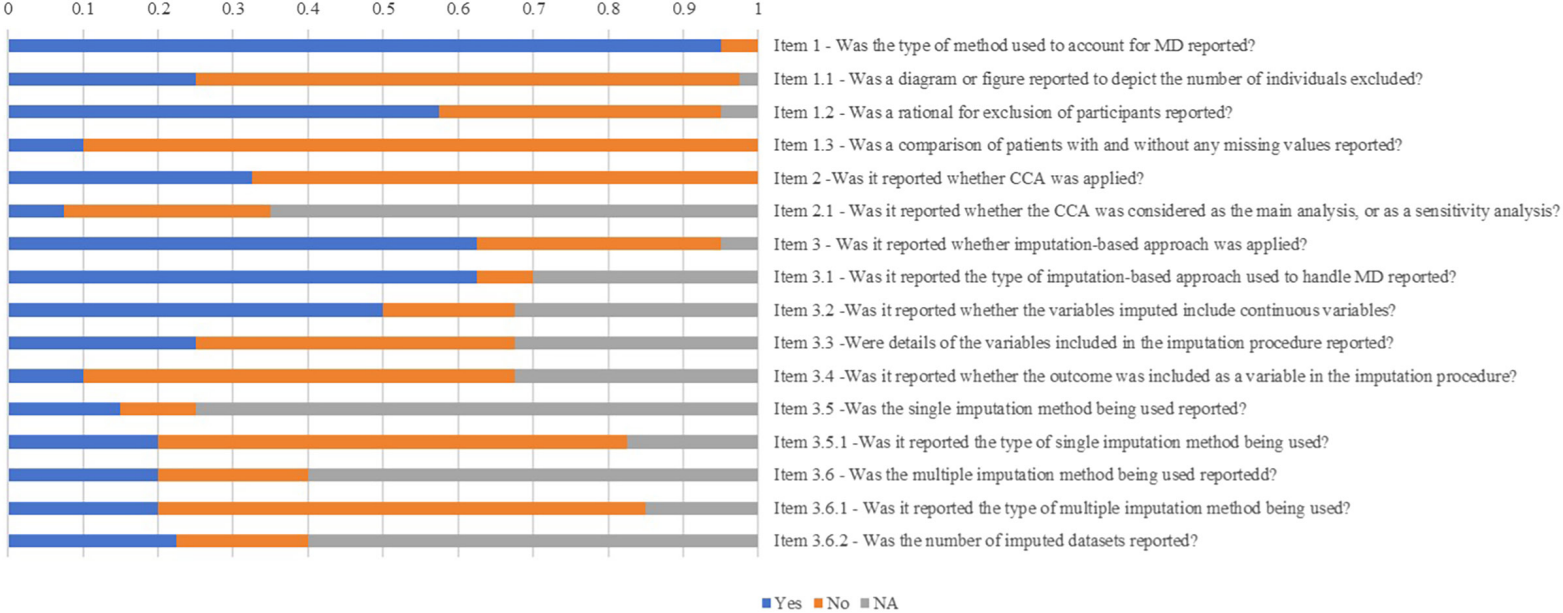
Missing data imputation approach at development stage of prediction model (*n* = 40).

In development studies, the most common approach for missing values was imputation (27/38) [5, 14, 15, 16, 18, 20, 21, 26, 27, 30, 32, 33, 35, 39, 40, 42, 43, 45, 47, 48, 49, 50, 53, 54, 57, 58, 62], followed by deletion (17/38) [13, 17, 20, 29, 30, 36, 37, 39, 43, 44, 46, 47, 51, 52, 53, 56, 61]. Simple imputation (SI) [15, 16, 18, 21, 27, 39, 49, 54, 57, 62] and multiple imputation (MI) [5, 14, 20, 26, 30, 32, 33, 35, 36, 40, 42, 43, 50, 53, 58] were reported in 10 and 15 studies, respectively, while one study utilized both SI and MI [48]. The last observation carried forward (*n* = 3) [27, 49, 62] was the most common method among studies using SI; the other methods included mean, median, and mode imputation. Multivariate imputation by chained equations (MICE) (6/15) was reported as the common MI method for addressing missing values [5, 26, 32, 48, 53, 58]. One study reported a fully conditional specification method [20]. Among studies that used deletion methods, 13 of the 17 articles applied CCA to handle missing data [13, 17, 20, 29, 30, 36, 37, 39, 43, 44, 46, 51, 61]. Four studies [39, 43, 44, 47] applied a machine learning method (two used random forest imputation [44, 47]) to address missing data (Figure 2).

#### 3.3.2 External Validation Stage

**FIGURE 3:**
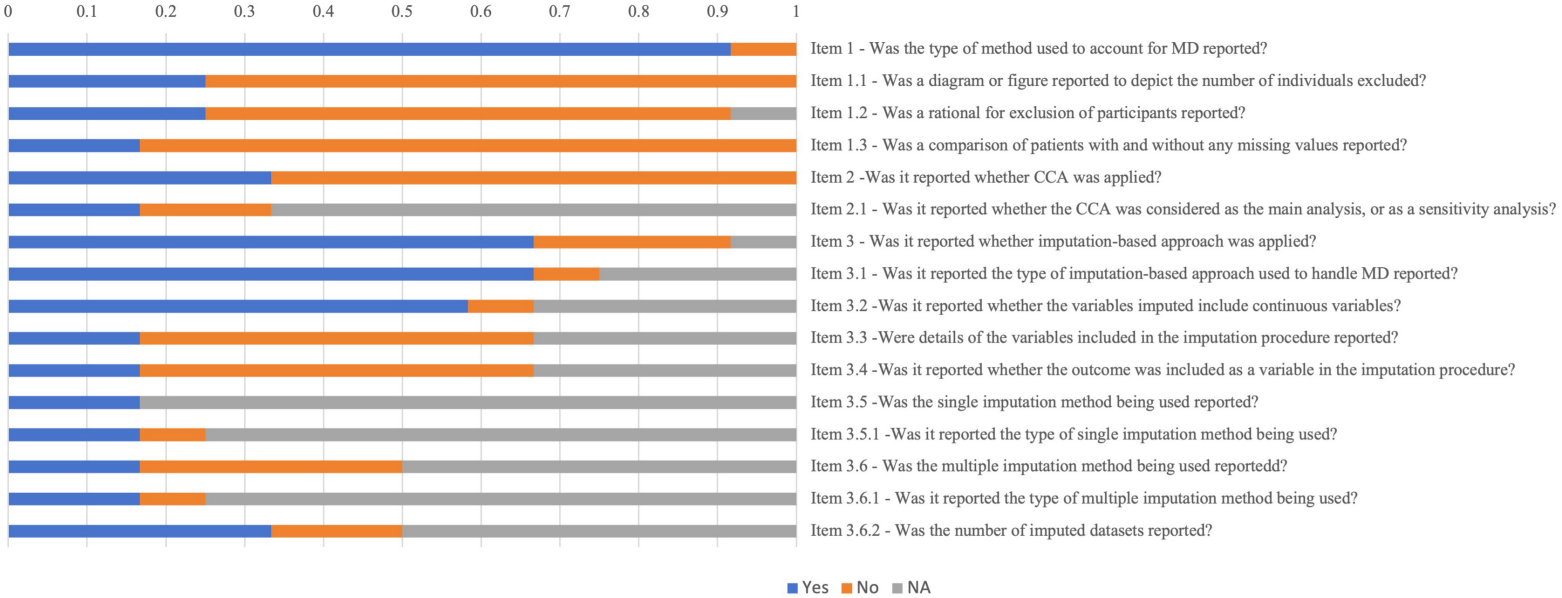
Missing data imputation approach at external validation stage of prediction model (*n* = 12).

Of the 11 studies with an external validation stage that reported missingness [14, 26, 27, 30, 39, 41, 42, 44, 50, 53, 58], six applied imputation methods to handle missing data [14, 27, 30, 42, 50, 53], three used deletion strategies [26, 44, 58], and one applied both methods [39]. Among the five articles that employed the MI strategy [14, 30, 42, 50, 53], only one reported the use of MICE [53]. Another study reported mean, median, and mode imputations [39]. In addition, CCA was applied in four studies that utilized the deletion approach [26, 39, 44, 58]. Six studies [14, 27, 39, 42, 44, 50] demonstrated a consistent approach to address missing data among the 11 studies [14, 26, 27, 30, 39, 41, 42, 44, 50, 53, 58] that included both development and external validation stages. Only two studies utilized imputation‐based methods during the development stage and opted for deletion strategies during the external validation stage [26, 58] (Figure 3).

### 3.4 Trends in Missing Data Processing Before and After 2015

An examination was conducted on the present status of reporting missing data and the modifications in strategies for handling missing data within the studies, including both pre‐ and post‐TRIPOD release. Until 2015, during the model development phase, nine [17, 18, 27, 30, 33, 45, 49, 61, 62] out of fifteen [17, 18, 23, 25, 27, 28, 30, 31, 33, 34, 45, 49, 59, 61, 62] studies reported missing data, with seven [18, 27, 30, 33, 45, 49, 62] utilizing imputation‐based techniques (including two employing MIs [30, 33]), two utilizing deletion methods [17, 61], and one combining both deletion and imputation approaches [30]. In 2016 and subsequent years, 31 [5, 13, 14, 15, 16, 20, 21, 24, 26, 29, 32, 35, 36, 37, 39, 40, 41, 42, 43, 44, 46, 47, 48, 50, 51, 52, 53, 54, 56, 57, 58] out of 36 [5, 13, 14, 15, 16, 19, 20, 21, 22, 24, 26, 29, 32, 35, 36, 37, 38, 39, 40, 41, 42, 43, 44, 46, 47, 48, 50, 51, 52, 53, 54, 55, 56, 57, 58, 60] reports contained missing data, with 15 [5, 14, 15, 16, 21, 26, 32, 35, 36, 40, 42, 50, 54, 57, 58] studies employing imputation‐based techniques (including 10 utilizing MI [5, 14, 26, 32, 35, 36, 40, 42, 50, 58]), eight utilizing deletion methods [13, 29, 37, 44, 46, 51, 52, 56], and five employing a combination of deletion and imputation strategies [20, 39, 43, 48, 53]. Regarding the external validation stage, a study of three papers published before 2016 revealed that two [27, 30] reported missing data and utilized imputation‐based methods. After 2016, 10 [14, 26, 39, 41, 42, 44, 50, 53, 54, 58] out of 13 [5, 14, 26, 29, 38, 39, 41, 42, 44, 47, 50, 53, 58] papers reported missing data, with four employing imputation‐based techniques [14, 42, 50, 53], three utilizing deletion methods [26, 44, 58], and one employing a combination of deletion and imputation strategies [39]. Among the papers that utilized imputation‐based methods for addressing missing data, four studies employed the MI approach [14, 42, 50, 53].

## 4 Discussion

Prediction models are being increasingly developed to support clinical decision‐making. Missing data is prevalent during the development and validation of prediction models, necessitating careful handling to prevent any negative impact on their quality. The present review's findings revealed that most (78.4%, 40/51) of the included studies on CVD risk among patients with T2DM reported missing data, and 74.5% (38/51) applied missing data handling approaches. Imputation was a commonly utilized approach, although most studies lacked detailed statistical information on the methods used. Notably, the frequency of missing data reports and the utilization of MI methods during the model development stage in the included articles demonstrated an increase subsequent to the publication of the TRIPOD guidelines. However, a comprehensive understanding is lacking regarding the proper selection and application of different methods, which may have implications for model development efficacy.

Missing predictor values pose obstacles to the development and practical implementation of predictive models, specifically affecting their predictive abilities. Interestingly, the missing data problem has garnered greater focus during the development stage than during the external validation and implementation stages [63, 64]. This phenomenon was also observed in our review, in which almost 78.4% (40/51) of included studies reported missing data during the development stage, while only 75% (12) reported them during the external validation stage. This difference may be associated with the lack of predictor variable management during the validation and implementation of a predictive model for novel patient populations.

Elucidating missing data characteristics is imperative to understanding data quality and making informed decisions regarding the selection of suitable data processing techniques. The missing data percentages and mechanisms are very important in evaluating the associated circumstances [65, 66]. In this review, only 22 studies that reported missing data included the overall number (*n* = 6) or missing data frequency (*n* = 16). The missing data percentage varied from < 1%–97.81%, showing great heterogeneity. This may be attributed primarily to the data sources utilized in developing the models' intricate nature, encompassing cohort, field survey data, and electronic medical records. Missing data mechanisms can be categorized as ‘missing completely at random (MCAR), MAR, or MNAR, depending on the reasons for missing data [67].

Analyzing missing data characteristics enables researchers to make informed decisions regarding the appropriate addressing strategies based on assumptions about data patterns. MCAR is rarely used and always involves negligible data, whereas scenarios involving MAR and MNAR are more likely. In this review, only two missingness mechanisms were reported, suggesting that many researchers continue to overlook the significance of accurately reporting missing data.

To correct the bias resulting from improper handling of missing data, TRIPOD emphasizes the significance of including missing data processing as a crucial study component [8]. As mentioned earlier, the rise in missing data reports and the use of MI methods in the model development phase post‐TRIPOD suggests that adherence to the TRIPOD guidelines may enhance the quality of prediction model studies, particularly in processing missing data. Evidence regarding missing data strategies dates to the 1970s, and many mature statistical methods have been proposed, including CCA and imputation‐based methods [68]. Processing relies on complete data and involves removing all cases with missing values to acquire a complete dataset for analysis. This method is user‐friendly and widely adopted by clinical researchers [68]. A previous systematic review of 152 prediction model studies utilizing machine learning suggested that the predominant method for addressing missing values is deletion (65/96), mostly via CCA (43/96), followed by imputation [69]. However, drawbacks include alterations in the original data distribution, potentially leading to an increase in bias, standard error, and imprecision in the regression coefficient estimates.

Imputation aims to predict missing values by obtaining values through the relationships within and between variables, including SI and MI. MI is the most popular missing data‐handling method in medical statistics. Our findings also indicated that the imputation‐based method was more widely used than the deletion strategies in both the development and external validation stages. This suggests that imputation‐based missing data approaches are progressively expanding and gaining acceptance among medical researchers, thereby broadening their scope. However, one study suggested that MI was more suitable when using research data, such as those from cohort studies that exhibit a low percentage of missing data and generally reasonable levels of MAR [11]. When using “real‐world” data, such as those from electronic health systems, missingness (typically informative and with a high rate using the MNAR mechanism), including missing indicators, could substantially improve predictive model performance [70, 71].

The application of the appropriate missing data handling method during the development and validation stages of predictive model is related to integrated missingness scenarios that consider missingness mechanisms, missingness patterns, and percentages of missing values. It indicates that researchers ought to comprehensively account for these factors when addressing missing values in predictive models. Compared with CCA, MI can reduce bias and enhance the model's predictive performance, particularly when the missing data mechanism is MAR. Other recently developed approaches address missing data by incorporating missing indicator variables, employing pattern‐mixing models, utilizing tree‐based integration, or applying machine learning techniques to circumvent inference challenges associated with missing data [64]. Furthermore, a lack of consistency in the strategies for addressing missing data during the development and validation stages can lead to an over or underestimation of the clinical prediction models [11, 72]. In this review, seven studies applied the same strategies to address missing data among the 13 studies that incorporated both development and external validation stages. In addition, among the studies with external validation, the details of the missing data strategies were fewer than those of model development. This may be attributed to the lack of emphasis on external validation and prediction model implementation, as evidenced by previous research findings [64]. The TRIPOD statement recommends the inclusion of information regarding the missing data percentage, missingness rationales, and notable distinctions between patients with complete versus incomplete data. Arguably, the reporting level is a critical foundation for evaluating the predictive model's efficacy. Provision of details is a crucial scientific research and reporting requirement to enable study replication. However, most studies that applied methods to handle missing data lacked sufficient detail. Thus, the methodological quality of predictive models must urgently be improved to promote good practices for handling missing data.

This review had some limitations. Some relevant studies might have been missed due to reliance on a single database. Nevertheless, we believe that the review comprehensively covers most of the literature through the application of a snowballing supplementary search. Furthermore, studies that were only in the external validation or deployment stages were not included. However, given that our study is built upon the same research framework that compared missing data processing strategies at various prediction model stages, its findings are more generalizable. Moreover, the analysis focused exclusively on models designed to predict CVD risk in patients with T2DM, which may differ from models used to predict CVD in other populations.

## 5 Conclusion

The issue of missing data in the development stage of CVD risk prediction models among patients with T2DM has gained much attention; however, insufficient consideration is paid to the validation stage. Notably, there is still room for improvement in selecting appropriate methods and providing details for handling missing data during the development and validation of CVD risk prediction models in T2DM patients. Additionally, the missingness mechanism should be carefully considered.

## Author Contributions

All the authors contributed to the conception or design of this study. Wenhui Ren, Keyu Fan, Zheng Liu, and Yanqiu Wu were involved in collecting and analyzing the data. Wenhui Ren, Keyu Fan, Huixin Liu, and Haiyan An wrote and prepared the manuscript. All the authors revised the manuscript critically and approved the final version. Additionally, Huixin Liu and Haiyan An are the guarantors, who had full access to all of the data in the study and took responsibility for the integrity of the data and the accuracy of the data analysis.

## Conflicts of Interest

The authors declare no conflicts of interest.

## Supporting information


**Appendix S1.** Supporting Information.

**Table S1.** Characteristics of the 51 included studies.
**Table S2.** Missing data details and handling strategies of the 51 included studies.

## Data Availability

The data that support the findings of this study are available from the corresponding author upon reasonable request.
