# Supplementary material for: Overcoming Missing Data: Accurately Predicting Cardiovascular Risk in Type 2 Diabetes, A Systematic Review
Source: J Diabetes. 2025 Jan 22;17(1):e70049. doi: 10.1111/1753-0407.70049 (PMC11753920; doi:10.1111/1753-0407.70049)
Supplement: Supplementary file 1 — Appendix S1. Supporting Information. [file JDB-17-e70049-s001.docx]

SUPPLEMENTARY APPENDIX

*Search strategy*

("diabetes mellitus, type 2"[MeSH Terms] OR ("Non-Insulin-Dependent Diabetes Mellitus"[Title/Abstract] OR "Type 2 Diabetes Mellitus"[Title/Abstract] OR "noninsulin dependent diabetes mellitus"[Title/Abstract] OR "noninsulin dependent diabetes mellitus"[Title/Abstract] OR "Type 2 Diabetes"[Title/Abstract]))

AND

("Cardiovascular Diseases"[MeSH Terms] OR ("Cardiovascular Disease"[Title/Abstract] OR "Major Adverse Cardiac Events"[Title/Abstract] OR "Cardiac Events"[Title/Abstract] OR "Cardiac Event"[Title/Abstract] OR "Adverse Cardiac Event"[Title/Abstract] OR "Adverse Cardiac Events"[Title/Abstract]))

AND

("Prediction model"[Title/Abstract] OR "Prediction Rules"[Title/Abstract] OR "Prognosis Models"[Title/Abstract] OR "Risk Scores"[Title/Abstract]
