# Supplementary material for: Overcoming Missing Data: Accurately Predicting Cardiovascular Risk in Type 2 Diabetes, A Systematic Review
Source: J Diabetes. 2025 Jan 22;17(1):e70049. doi: 10.1111/1753-0407.70049 (PMC11753920; doi:10.1111/1753-0407.70049)
Supplement: Supplementary file 2 — Table S1. Characteristics of the 51 included studies. Table S2. Missing data details and handling strategies of the 51 included studies. [file JDB-17-e70049-s002.docx]

**Table S1 Characteristics of the 51 included studies**

| **First author/ Publication year** | **Study designs** | **Data source** | **Study population [sample size]** | **Outcomes** | **Model types** | **Stage of prediction model** |
| --- | --- | --- | --- | --- | --- | --- |
| Stevens, R. J. et al 2001 | Cohort Study | UKPDS | Participants with newly diagnosed T2DM aged 25–65 years [n=4540] | CHD (defined as the occurrence of fatal or non-fatal MI or sudden death, verified by two independent clinical assessors) | Cox proportional hazards regression model | Development |
| Nakatou, T. et al 2004 | Cross-Sectional Study | The diabetic centre of Okayama Saiseikai General Hospital from 1 December 1997 to 31 January 2002 | Participants with T2DM were hospitalised for the treatment of diabetes [n=342] | Atherosclerosis | Logistic regression | Development |
| Jiang, R. et al 2004 | Cohort Study | The Health Professionals’ Follow-up Study in 1986 | Participants with diabetes who were aged 46–81 years [n=746] | Hospital discharge diagnoses and mortality | Cox proportional hazards regression model | Development |
| Yang, X. et al 2007 | Cohort Study | The Prince of Wales Hospital from 1995 to 2005 | Hong Kong Chinese T2DM participants without a history of stroke at baseline [n=7920] | Diagnosed IHD | Cox proportional hazards regression model | Development |
| Yang, X. et al 2008 | Cohort Study | The Prince of Wales Hospital from 1995 to 2005 | Chinese participants with type 2 diabetes who were free of past history of HF and CHD [n=7067] | Time to first fatal or non-fatal cardiovascular event | Cox proportional regression analysis | Development & Internal Validation |
| Maracy, M. R. et al 2008 | Cross-Sectional Study | IEMRC from 2001 to 2005 | Participants with type 2 diabetes with or without IHD [n=2101] | The first 4.5 years cardiovascular risk prediction | Logistic regression model; Risk scores | Development |
| Davis, W. A. et al 2010 | Cross-Sectional Study | **【Development】** From Western Australia from 1993 to 1996  **【Validation】** BHS comprises serial population from 1993 to 1996 | Type 2 diabetes participants [n=1240/180] | Annual incidence of death or diabetes complications (MI, stroke, IHD, CHF, amputation, blindness, renal failure and ulcer), including second events for MI, stroke and amputation, life expectancy and quality-adjusted life years (QALYs) | Cox proportional hazards regression model | Development & External Validation |
| Elley, C. R. et al 2010 | Cohort Study | DCS in New Zealand from 2000 to 2006.  **【Development】** From the north of New Zealand  **【Validation】** From the south of New Zealand. | Participants with type 2 diabetes determined by their primary-care physician, had commenced the “Get Checked” program, and had all risk variables recorded during the first assessment or within 2 years [n=36127] | Fatal and non-fatal CHD and CVD | Cox proportional hazards regression models | Development & External Validation |
| Kengne, A. P. et al 2011 | RCT | ADVANCE | Participants diagnosed with diabetes at the age of 30 years or older, at least 55 years of age at entry, be atincreased risk of vascular events [n=7168] | Incident heart failure; separate risk equations in men and women for evaluation at 10 years | Cox proportional hazards regression model | Development |
| Raffield, L. M. et al 2013 | Cohort Study | DHS in Western North Carolina from 1998 to 2005 | Self-described European American participants with T2DM from DHS families [n=983] | A major vascular event, defined as a major CVD event (non-fatal MI, non-fatal stroke or death from any cardiovascular cause) or a major renal event (renal death or requirement for renal replacement therapy, defined as dialysis or renal transplantation) | Cox proportional hazards regression model | Development & Internal Validation |
| Hayes, A. J. et al 2013 | Cohort Study | UKPDS | Participants with newly diagnosed type 2 diabetes mellitus, aged 25–65 years [n=4031] | Cardiovascular‑death | Semi-parametric proportional hazards survival models | Development & Internal Validation |
| Adams, J.N. et al 2014 | Cohort Study | DHS | Siblings with T2DM and without advanced nephropathy were recruited, with unaffected siblings [n=1208] | Cerebrovascular hospitalisation within two years and cerebrovascular re-hospitalisation within 90 days of the previous cerebrovascular hospitalisation | Cox proportional hazards regression model | Development |
| Yeboah, J. et al 2014 | Retrospective Cohort Study | MESA from 2000 to 2003 | Caucasians aged 45-75 from three neighbouring cities in the metropolitan Ruhr area of Germany in a single centre [n=1343] | The 5-year CVD risk prediction models and tools for Chinese primary care patients with T2DM | Cox model conditional model | Development & Internal Validation |
| Piniés, J. A. et al 2014 | Prospective Cohort Study | Sentinel Practice Network from 1998 to 2000 | Participants older than 24 years of age with type 2 diabetes [n=777] | Ascertain IHD and cerebrovascular disease | Cox proportional hazards regression model | Development & Internal Validation |
| Hippisley-Cox, J. et al 2015 | Cohort Study | The QResearch and CPRD databases from 1 January 1998 to 31 July 2014 | Participants with diabetes aged 25–84 years registered with eligible practices [n=437806/137028+197 905] | Stroke | Cox proportional hazards models; risk prediction equations | Development & External Validation |
| Woodward, M. et al 2016 | RCT | ADVANCE | Participants recruited from 20 countries, were found to be representative of general diabetes populations [n=7301] | All-cause and expanded CV specific death status | Cox proportional hazards regression model | Development |
| Sadeghpour, S. et al 2016 | Cohort Study | In Isfahan from 1992 to 2004 | Men and women over the age of 35 who present with T2DM [n=2638] | The first composite CV event | Cox cause‑specific hazards model and Fine and Gray model based on subdistribution hazards model | Development |
| Wan, E.Y.F. et al 2017 | Retrospective Cohort Study | Chinese patients managed in public primary care clinics | Chinese participants clinically diagnosed with T2DM, aged between 18 and 79 years, managed in public primary care clinics and without past history of CVD or end stage renal disease [n=137935] | CVD (defined as any hospital admission or death from non-fatal MI, stroke, HFC, cerebrovascular diseases, or transient cerebral ischaemic attacks and related syndromes between baseline and December 31, 2017) | Cox proportional hazards regression model; decision tree | Development |
| Basu, S. et al 2017 | RCT | **【Development】**ACCORD from 2001 to 2009 (patients had both microvascular and cardiovascular outcomes)  **【Validation】**Look AHEAD from 2001 to 2012 (patients only cardiovascular event) | Participants were aged 40–79 years with type 2 diabetes and had an HbA1c of at least 7·5% (57 mmol/mol), and either history of cardiovascular disease or risk factors for cardiovascular disease [n=9635/4760] | Predict 3-year ASCVD risk (ASCVD was defined as any of the CHD, cerebrovascular disease or peripheral vascular disease) | Cox proportional hazards regression model | Development & External Validation |
| Oellgaard, J. et al 2018 | Cohort Study Based RCT | Steno-2 study from 1995 to 2014 | Participants with diabetes exhibiting persistent microalbuminuria [n=151] | CHD and a composite endpoint of incident death of CVD, MI or revascularisation, stroke, amputation or peripheral revascularisation | Cox proportional hazards regression model; logistic models | Development |
| Alrawahi, A. H. et al 2018 | Retrospective Cohort Study | Three polyclinics (Nizwa,Bahla and Izki Polyclinics) and one large health centre (Manah Health Centre) were selected as the study institutions from September 2015 to July 2016 | Omanis with type 2 diabetes residing in the Aldakhiliyah Governorate (Province) [n=2039] | 5-year CV risk | Cox proportional hazards regression model | Development |
| Dahai, Y. et al 2018 | Two Prospective Cohort Study | **【Development】**18 general practices across Cambridgeshire, England, in 2008/2009 with linkage to inpatient hospitalisation  **【Validation】**RAPSID | Participants with type 2 diabetes [n=4704/1121] | Cerebrovascular hospitalisation within two years and cerebrovascular re-hospitalisation within 90 days of the previous cerebrovascular hospitalisation | Logistic regression | Development & External Validation |
| Wan, E.Y.F. et al 2018 | Retrospective Cohort Study | The 74 general out-patient clinics of the Hong Kong Hospital Authority from 1 January 2010 to 31 December 2010 | Chinese participants aged 18-79 clinically diagnosed with T2DM [n=137935] | The 5-year CVD risk prediction models and tools for Chinese primary care patients with T2DM | Linear, logistic or ordered logistic regression | Development & Internal Validation |
| Li, T. C. et al 2018 | Retrospective Cohort Study | TDS enrolled in NDCMP from 2001 to 2004 | Chinese Participants with type 2 diabetes [n=28124] | Ascertain IHD and cerebrovascular disease | Cox proportional hazards regression model | Development & Internal Validation |
| Nowak, C. et al 2018 | Prospective Cohort Study | 25 primary healthcare centres in the counties of Östergötland and Jönköping, Sweden, from November 2005 to December 2008 | Participants aged 55–65 years with type 2 diabetes [n=1211] | Stroke | Cox proportional hazards regression model | Development |
| Berg, D. D. et al 2019 | RCT | The SAVOR-TIMI 53 and DECLARE-TIMI 58 trials | **【Development】P**articipants that 79% with established CVD and 21% with multiple risk factors for CVD [n=8212]  **【Validation**】Participants that 41% with established CVD and 59% with multiple risk factors for CVD [n=8578] | All-cause and expanded CV specific death status | Cox proportional hazards regression model | Development & External Validation |
| Mei, J. and E. Xia et al 2019 | Cohort Study | The EHR repository | Participants with at least one diabetes-related diagnosis and one diabetes-related prescription [n=4143] | The first composite CV event | Knowledge Learning Symbiosis | Development |
| Segar, M. W. et al 2019 | Cohort Study | **【Development】**ACCORD  **【Validation】**ALLHAT | **【Development】**Participants had established ASCVD or were 55–79 years of age with anatomic evidence of atherosclerosis, albuminuria, left ventricular hypertrophy, or two or more other cardiovascular risk factors(current smoking, hyperlipidaemia, hypertension, or obesity) [n=10251]  **【Validation】**New-onset HF [n=10819] | CVD (defined as any hospital admission or death from non-fatal MI, stroke, HF, cerebrovascular diseases, or transient cerebral ischaemic attacks and related syndromes between baseline and December 31, 2017) | Random survival forest (RSF) methods | Development & External Validation |
| Berkelmans, G.F.N. et al 2019 | Cohort Study | NDR and SCID | Participants aged >18 years with a diagnosis of T2DM with or without prevalent CVD [n=389366/197785] | Predict 3-year ASCVD risk (ASCVD was defined as any of CHD, cerebrovascular disease or peripheral vascular disease) | Two complementary competing risk-adjusted Cox proportional hazards functions | Development & Internal Validation |
| Quan, J. et al 2019 | Retrospective Cohort Study | **【Development】** Hong Kong Hospital Authority health records from 2006 to 2014  **【Validation】**Singapore Ministry of Health records from 2008 to 2016 | Adults aged 20 years or over with type 2 diabetes [n=678750/386425] | Ascertain IHD and cerebrovascular disease | Cox proportional hazards models; risk scores | Development & External Validation |
| Kim, M. K. et al 2020 | Cohort Study | The National Health Insurance Database in Korea from 2009 to 2012 | Participants aged 40–64 years with type 2 DM who received a health examination [n=1297131] | 5-year risk of stroke | Cox proportional hazards regression model | Development & Internal Validation |
| Williams, B. A. et al 2020 | Retrospective Cohort Study | EMR data warehouse of a single integrated healthcare delivery system in the northeast United States from January 1, 2001 to November 10, 2015 | From primary care and other healthcare services through the study institution for at least 2 years [n=54452] | A new HHF (defined as an EMR documented, post-index date hospital admission with HF as the primary diagnosis in the absence of any prior documented HF diagnosis which constituted an exclusion criterion) | Cox proportional hazards regression model | Development |
| Xue, M. et al 2020 | Cross-Sectional Study | A large community survey in Urumqi in 2018 | T2DM participants aged over 20 [n=30507] | Diagnosis of hypertension | Logistic regression | Development & Internal Validation |
| Yang, P. et al 2020 | RCT | ACCORD | Participants with T2DM aged 40 to 79 years with high risk for CVD [n=9240] | Incident AF (defined as the combined outcome of atrial fibrillation and atrial flutter). Time to incident AF was defined as the time between the baseline visit and the visit first detected AF. | Cox proportional hazards regression model | Development |
| Shi, R. et al 2020 | Cohort Study | In the seven communities including Huamu, Jinyang, Sanlin, Siping, Yinhang, Daqiao, and Jiangpu in Shanghai from September 2014 to September 2019. | Participants with T2DM with a history of stroke [n=4335] | Stroke | The multivariate logistic regression analysis. | Development & Internal Validation |
| Lee, S. H. et al 2020 | Cohort Study | NHIS from 2009 to 2012 | Participants with T2DM (<40 or ≥65 years) [n=1272992] | MI (defined as when ICD-10 codes I21 or I22 were newly recorded during hospitalisation). The study population was followed-up by the date of MI, or until 31 December 2017, whichever came first. The mean follow-up period was 7.1±1.2 years. | Cox proportional hazards regression model | Development & Internal Validation |
| Schiborn, C. et al 2021 | Prospective Cohort Study | **【Development】** EPIC-Potsdam cohort from 1994 to 2012  **【Validation】** EPIC-Heidelberg from 1994 to 2012 | **【Development】** age mainly 35–65 years, 60.4% women [n=25933]  **【Validation】**age 35–66 years, 53.3% women [n=23529] | 10‑year cardiovascular disease risk | Cox proportional hazards regression model;  Fine and Gray model | Development & External Validation |
| Liu, C. S. et al 2021 | Retrospective Cohort Study | The DCMP of the CMUH in Taiwan, set up in 2002 | Participants with a clinically confirmed diagnosis of DM based on the criteria of American Diabetes Association (International Classification Disease, Ninth Revision, Clinical Modification, abbreviated as ICD-9-CM; Code of 250) [n=9692] | All-cause and expanded CV specific death status | Cox proportional hazards regression model | Development & Internal Validation |
| Caplan, E. O. et al 2021 | Retrospective Cohort Study | the Humana from January 1, 2011, to December 31, 2018 | Participants with T2D aged 65-89 years [n=362791] | The first composite CV event | Risk score model | Development |
| Lee, S. et al 2021 | Cohort Study | The government-funded hospitals and clinics in Hong Kong from January 1, 2009 to December 31, 2009 | Participants aged above 40 had documented diagnosis of T2DM under the International Classification of Disease, Ninth Edition (ICD-9) coding system, or prescribed anti diabetic agents ,without prior history of AMI and SCD episodes [n=261308] | The time to the initial AMI and non-AMI-related SCD episode(defined as days from 1 January 2009 to the date of initial AMI/ non-AMI-related SCD or the day of 31 December 2019 | Cox proportional hazards regression model | Development |
| Williams, B. A. et al 2021 | Retrospective Cohort Study | An integrated healthcare system and EMRs | Participants with T2DM followed for an average of 7.5 years [n=59180] | CV-related death | Cox cause‑specific hazards model; risk scores | Development |
| Chu, H. et al 2021 | Cross-Sectional Study | The Department of Endocrinology, Affiliated Hospital of Harbin Medical University Harbin from 2017 to 2020. | Participants with T2DM [n=834] | CVD | Deep Neural Network | Development & Internal Validation |
| Lin, Y. et al 2022 | **【Development】:**A Randomised, Multicentre Study;  **【Validation】:** A Long-Term, Randomised, Double Blind, Placebo-Controlled Study | **【Development】**ACCORD  **【Validation】**The Harmony Outcome trial and CRIC | Participants with T2DM and without HF history [n=9649/7436+1996] | HHF [defined as hospitalisation for congestive HF (with documented clinical and radiological evidence)] | Dynamic Cox model | Development & External Validation |
| Wan, C. et al 2022 | Prospective Cohort Study | **【Development】** The OMOP CDM platform in the First Affiliated Hospital of NMU, from January 1, 2008 to December 31, 2017  **【Validation】** The SCI, from January 1, 2008 to December 31, 2017 | Participants diagnosed with T2DM [n=84630/119891] | CVD [defined as any hospital admission or death from non-fatal MI (International Classification of Disease [ICD-10] codes I21–I22), stroke (ICD-10 codes I60–I69), HF (ICD-10 code I50), cerebrovascular diseases, or transient cerebral ischaemic attacks and related syndromes (ICD-10 codes G45)] | Cox proportional hazards regression model | Development & External Validation |
| SCORE2-Diabetes Working Group and the ESC Cardiovascular Risk Collaboration. et al 2023 | Cohort Study | **【Development】**SCID, CPRD, UKB and ERFC;  **【Validation】**SNDR, SIDIAP and EUBIROD | Participants with diabetes [n=229460/216980] | CVD (defined as a composite of CV mortality, non-fatal MI, and non-fatal stroke) | Sex-specific competing risk-adjusted models | Development & External Validation |
| Ding, J.Y. et al 2023 | Cohort study | 3Bext database (the extending study of the 3B study19), containing admitted to 94 tier 1 to tier 3 hospitals across China from 2010 to 2014 | Type 2 diabetes mellitus patients admitted to 94 tier 1 to tier 3 hospitals across China [n=719/278] | Predict 3-year ASCVD risk (ASCVD was defined by any of CAD, cerebrovascular disease or peripheral vascular disease] | Logistic regression, support vector machine, gradient boosting decision tree (GBDT), random forest (RF) and adaptive boosting (AdaBoost) | Development & External Validation |
| Hao, M. et al 2023 | Cross-sectional study | The First Affiliated Hospital of Shenzhen University from January 2014 to December 2021 | T2DM patients [n=3030] | Diagnosed with DCM based on Doppler echocardiography in T2DM patients with diastolic dysfunction | Multivariable logistic regression analysis | Development & Internal Validation |
| Said, F. et al 2024 | RCT | **【Development】**The Aliskiren Trial in Type 2 Diabetes Using Cardiorenal Endpoints (ALTITUDE)  **【Validation】**The placebo arm of the Canagliflozin Cardiovascular Assessment Study (CANVAS) | Patients with T2D and evidence of albuminuria or cardiovascular disease [n=8561/4330] | New-onset HF was defined as an unplanned first-time hospitalisation for HF in patients who did not have a history of HF at baseline | Cox proportional hazard models | Development & External Validation |
| Shah, B.R. et al 2024 | Cohort study | **【Development】**Electronic medical record (EMR) data in Ontario from April 1991 to March 2016  **【Validation】**Administrative and EMR data in the province of Manitoba from April 1991 to March 2016 | T2DM [n=25088/11,416] | Major clinically important microvascular events, CVD events, and mortality | Cox proportional hazard model, Fine–Gray competing risk model, Logistic regression, linear regression | Development & External Validation |
| Tusongtuoheti, X. et al 2024 | Cohort study | Zhenhai Lianhua Hospital, Ningbo, China, from January 2018 to December 2022 | Participants aged ≥ 18 years who either self-report T2DM, are undergoing pharmacological treatment for T2DM, or meet the diagnostic criteria of T2DM [n=3084] | SCAS was defined as CIMT > 1.0 mm and/or the presence of plaque without clinical manifestations | Linear discriminant analysis, logistic regression, Least absolute shrinkage and selection operator (LASSO), random forest-recursive feature elimination (RF-RFE),linear discriminant analysis (LDA), logistic regression (LR), Naive Bayes (NB), random forest (RF), support vector machine (SVM), and extreme gradient boosting (XGboost) | Development & Internal Validation |
| Sang, H. et al 2024 | Cohort study | **【Development】**A tertiary hospital at the Kyung Hee University Medical Center from January 1, 2008, to December 31, 2022  **【Validation】**The secondary hospitals Kyung Hee University Medical Center at Gangdong and Gachon University Gil Hospital " | T2DM [n=12809/2019] | Ischaemic heart disease and myocardial infarction, heart failure, and atrial fibrillation | Decision-tree-based ensemble models, such as the XGBoost (XGB), random forest (RF), LightGBM (LGM), and AdaBoost (ADB), and linear classification models, such as logistic regression (LR) and support vector machine (SVM) | Development & External Validation |

**Abbreviations:** ACCORD, Action to Control Cardiovascular Risk in Diabetes study; ADVANCE, Action in Diabetes and Vascular disease: preterax and diamicron-MR controlled valuation; AF, atrial fibrillation; ALLHAT, the Antihypertensive and Lipid-Lowering Treatment to Prevent Heart Attack Trial; ASCVD, atherosclerotic cardiovascular disease; CAD, coronary artery disease; CHD, Coronary heart disease; CMUH, the China Medical University Hospital; CPRD, Clinical Research Practice Datalink; CRIC, The Chronic Renal Insufficiency Cohort; CV, Cardiovascular; DCM, diabetic cardiomyopathy; DCMP, Diabetes Care Management Program; DCS, The Diabetes Cohort Study; DHS, The Diabetes Heart Study; EMR, Electronic medical record; EPIC, The European Prospective Investigation into Cancer and Nutrition; ERFC, Emerging Risk Factors Collaboration; EUBIROD, European Best Information through Regional Outcome in Diabetes; HF, heart failure; HHF, hospitalisation for heart failure; IEMRC, Isfahan Endocrine and Metabolism Research Centre; IHD , ischaemic heart disease; Look AHEAD, The Action for Health in Diabetes; MESA, Multi-Ethnic Study of Atherosclerosis; MI, Myocardial Infarction; NDCMP, the National Diabetes Care Management Program; NDR, The Swedish National Diabetes Registry; NHIS, The Korean National Health Insurance Service; NMU, Nanjing Medical University; OMOP CDM, Observational Medical Outcomes Partnership Common Data Model; RAPSID, the RAndomized controlled trial of Peer Support in type 2 Diabetes; RCT, Randomised controlled trial; SCAS, subclinical atherosclerosis; SCD, sudden cardiac death; SCI, Scottish Care Information; SCID, Scottish Care Information—Diabetes; SIDIAP, Sistema d’Informació per al Desenvolupament de la Investigació en Atenció Primària; SNDR, Swedish National Diabetes Register; T2DM, diabetes mellitus type 2; TDS, Taiwan Diabetes Study; UKB, UK Biobank; UKPDS, United Kingdom Prospective Diabetes Study.

**Table S2 Missing data details and handling strategies of the 51 included studies**

| **First author/Publication year** | **Stage of prediction model** | **Reported missing data** | **Percentage of missing data** | **Mechanism of missing data** | **Missing data processing strategy** | **Missing data methods** |
| --- | --- | --- | --- | --- | --- | --- |
| Stevens, R. J. et al 2001 | Development stage | Yes | 4.80% | NR | Deletion | |
| Nakatou, T. et al 2004 | Development stage | No | NA | NA | NA | NA |
| Jiang, R. et al 2004 | Development stage | Yes | NR | NR | Imputation-based approach | Single imputation |
| Yang, X. et al 2007 | Development stage | No | NA | NA | NA | NA |
| Yang, X. et al 2008 | Development stage | No | NA | NA | NA | NA |
| Maracy, M. R. et al 2008 | Development stage | No | NA | NA | NA | NA |
| Davis, W. A. et al 2010 | Development stage | Yes | 66.67% | MAR | Deletion | |
|  | External validation stage | No | NA | NA | NA | NA |
| Elley, C. R. et al 2010 | Development stage | Yes | NR | NR | Imputation-based approach | Single imputation |
|  | External validation stage | Yes | NR | NR | Imputation-based approach | Single imputation |
| Kengne, A. P. et al 2011 | Development stage | No | NR | NR | NR | NR |
| Raffield, L. M et al 2013 | Development stage | Yes | NR | NR | Imputation-based approach | Single imputation |
| Hayes, A. J. et al 2013 | Development stage | Yes | NR | NR | Imputation-based approaches | LOCF |
| Adams, J.N. et al 2014 | Development stage | Yes | NR | NR | Imputation-based approaches | Single imputation |
| Yeboah, J. et al 2014 | Development stage | Yes | <4% | NR | Imputation-based approach | Multiple imputation |
| Piniés, J. A. et al 2014 | Development stage | No | NA | NA | NA | NA |
| Hippisley-Cox, J. et al 2015 | Development stage | Yes | NR | NR | Deletion; Imputation-based approach | CCA; Multiple imputation |
|  | External validation stage | Yes | NR | NR | Imputation-based approach | Multiple imputation |
| Woodward, M. et al 2016 | Development stage | Yes | 4.79%**†** | NR | Deletion | |
| Sadeghpour, S. et al 2016 | Development stage | Yes | NR | NR | Deletion | |
| Wan, E.Y.F. et al 2017 | Development stage | Yes | NR | NR | NR | NR |
| Basu, S. et al 2017 | Development stage | Yes | 6%**†** | NR | Deletion | |
|  | External validation stage | No | NA | NA | NA | NA |
| Oellgaard, J. et al 2018 | Development stage | No | NA | NA | NA | NA |
| Alrawahi, A. H. et al 2018 | Development stage | No | NA | NA | NA | NA |
| Dahai, Y. et al 2018 | Development stage | Yes | 3.17%~14.56% | NR | Imputation-based approach | Multiple imputation |
|  | External validation stage | Yes | <1% | NR | Deletion | |
| Wan, E.Y.F. et al 2018 | Development stage | Yes | 21~33% | NR | Imputation-based approach | Multiple imputation |
| Li, T. C. et al 2018 | Development stage | Yes | NR | NR | Imputation-based approach | Multiple imputation |
| Nowak, C. et al 2018 | Development stage | Yes | NR | NR | Deletion; Imputation-based approach | Multiple imputation |
| Berg, D. D. et al 2019 | Development stage | No | NA | NA | NA | NA |
|  | External validation stage | No | NA | NA | NA | NA |
| Mei, J. and E. Xia et al 2019 | Development stage | Yes | 92.4% | NR | Deletion | |
| Segar, M. W. et al 2019 | Development stage | Yes | >10%**†** | NR | Non-imputation based approaches;  Imputation-based approach; Deletion | A random forest imputation, Deletion |
|  | External validation stage | No | NA | NA | NA | NA |
| Berkelmans, G.F.N. et al 2019 | Development stage | Yes | 0~ 43% | NR | Imputation-based approach | Single imputation |
| Quan, J. et al 2019 | Development stage | Yes | 14.42~73.69% | MNAR | Imputation-based approach | Multiple imputation |
|  | External validation stage | Yes | NR | NR | Deletion | |
| Kim, M. K. et al 2020 | Development stage | Yes | NR | NR | Deletion | |
| Williams, B. A. et al 2020 | Development stage | Yes | NR | MNAR | Imputation-based approach | Single imputation |
| Xue, M. et al 2020 | Development stage | Yes | NR | NR | Imputation-based approach | Single imputation |
| Yang, P. et al 2020 | Development stage | Yes | NR | NR | Imputation-based approach | Multiple imputation |
| Shi, R. et al 2020 | Development stage | Yes | NR | NR | Deletion | |
| Lee, S. H. et al 2020 | Development stage | Yes | 45.85%**†** | NR | Deletion | CCA |
| Schiborn, C. et al 2021 | Development stage | Yes | 0~97.81% | NR | Imputation-based approach | MICE |
|  | External validation stage | Yes | 0~89.59% | NR | Imputation-based approach | MICE |
| Liu, C. S. et al 2021 | Development stage | Yes | 30% | NR | Imputation-based approach; Deletion | Multiple imputation |
| Caplan, E. O. et al 2021 | Development stage | Yes | 0.5-85.9% | NR | Imputation-based approach | Multiple imputation |
| Lee, S. et al 2021 | Development stage | No | NR | NR | NR | NR |
| Williams, B. A. et al 2021 | Development stage | Yes | NR | NR | Imputation-based approach | Single imputation |
| Chu, H. et al 2021 | Development stage | No | NR | NR | NR | NR |
| Lin, Y. et al 2022 | Development stage | Yes | NR | NR | Imputation-based approach | Multiple imputation |
|  | External validation stage | Yes | >70% | NR | Imputation-based approach | Multiple imputation |
| Wan, C. et al 2022 | Development stage | Yes | 2.44% | NR | Deletion; Imputation-based approach | Multiple imputation |
|  | External validation stage | Yes | NR | NR | Imputation-based approach | Multiple imputation |
| Ding, J.Y. et al 2023 | Development stage | Yes | 0.11 | NR | Imputation-based approach; Deletion | Single imputation; CCA |
|  | External validation stage | Yes | 0.09 | NR | Imputation-based approach; Deletion | Single imputation; CCA |
| SCORE2-Diabetes Working Group and the ESC Cardiovascular Risk Collaboration. et al 2023 | Development stage | Yes | 0~56.3% | NR | Imputation-based approach | Multiple imputation |
|  | External validation stage | Yes | NR | NR | Imputation-based approach | Multiple imputation |
| Hao, M. et al 2023 | Development stage | Yes | 0.8%~20.5% | MAR | Imputation-based approach | Multiple imputation |
| Said, F. et al 2024 | Development stage | Yes | NR | NR | NR | NA |
|  | External validation stage | Yes | NR | NR | NR | NA |
| Shah, B.R. et al 2024 | Development stage | Yes | 18.5%~ 49.4% (66%**†**) | NR | Imputation-based approach | Multiple imputation |
|  | External validation stage | Yes | 100%**†** | NR | Imputation-based approach | Multiple imputation |
| Tusongtuoheti, X. et al 2024 | Development stage | Yes | 2.62%**†** | NR | Imputation-based approach; Deletion | Multiple imputation |
| Sang, H. et al 2024 | Development stage | Yes | 81.17%**†** | NR | Deletion | CCA |
|  | External validation stage | Yes | 89.32%**†** | NR | Deletion | CCA |

**† Percentage of missing data based on individual statistics.**

**Abbreviations:** CCA, Complete Case Analysis; MICE, Multiple imputation by chained equations; NA, Not Applicable; NR, Not Reported; LOCF, Last Observation Carries Forward.
